# Striatal Volume Increase After Six Weeks of Selective Dopamine D_2/3_ Receptor Blockade in First-Episode, Antipsychotic-Naïve Schizophrenia Patients

**DOI:** 10.3389/fnins.2020.00484

**Published:** 2020-05-20

**Authors:** Helle G. Andersen, Jayachandra M. Raghava, Claus Svarer, Sanne Wulff, Louise B. Johansen, Patrick K. Antonsen, Mette Ø. Nielsen, Egill Rostrup, Anthony C. Vernon, Lars T. Jensen, Lars H. Pinborg, Birte Y. Glenthøj, Bjørn H. Ebdrup

**Affiliations:** ^1^Center for Clinical Intervention and Neuropsychiatric Schizophrenia Research and Center for Neuropsychiatric Schizophrenia Research, Mental Health Centre Glostrup, University of Copenhagen, Glostrup, Denmark; ^2^Department of Clinical Medicine, Faculty of Health and Medical Sciences, University of Copenhagen, Copenhagen, Denmark; ^3^Functional Imaging Unit, Department of Clinical Physiology, Nuclear Medicine and PET, University of Copenhagen, Glostrup, Denmark; ^4^Neurobiology Research Unit, Department of Neurology, Rigshospitalet, University of Copenhagen, Copenhagen, Denmark; ^5^Department of Basic and Clinical Neuroscience, Institute of Psychiatry Psychology and Neuroscience, King’s College London, London, United Kingdom; ^6^Medical Research Council Centre for Neurodevelopmental Disorders, King’s College London, London, United Kingdom; ^7^Department of Clinical Physiology and Nuclear Medicine, Herlev Hospital, University of Copenhagen, Herlev, Denmark

**Keywords:** schizophrenia, dopamine receptor, first-episode antipsychotic-naïve, striatum, SPECT, sMRI, antipsychotic drug, longitudinal

## Abstract

Patients with chronic schizophrenia often display enlarged striatal volumes, and antipsychotic drugs may contribute via the dopamine D_2/3_ receptor (D_2/3_R) blockade. Separating the effects of disease from medication is challenging due to the lack of a proper placebo-group. To address this, we conducted a longitudinal study of antipsychotic-naïve, first-episode schizophrenia patients to test the hypothesis that selective blockade of D_2/3_R would induce a dose-dependent striatal volume increase. Twenty-one patients underwent structural magnetic resonance imaging (sMRI), single-photon emission computed tomography (SPECT), and symptom severity ratings before and after six weeks of amisulpride treatment. Twenty-three matched healthy controls underwent sMRI and baseline SPECT. Data were analyzed using repeated measures and multiple regression analyses. Correlations between symptom severity decrease, volume changes, dose and receptor occupancy were explored. Striatal volumes did not differ between patients and controls at baseline or follow-up, but a significant group-by-time interaction was found (*p* = 0.01). This interaction was explained by a significant striatal volume increase of 2.1% in patients (Cohens *d* = 0.45). Striatal increase was predicted by amisulpride dose, but not by either D_2/3_R occupancy or baseline symptom severity. A significant reduction in symptom severity was observed at a mean dose of 233.3 (SD = 109.9) mg, corresponding to D_2/3_R occupancy of 44.65%. Reduction in positive symptoms correlated significantly with striatal volume increase, driven by reductions in hallucinations. Our data demonstrate a clear link between antipsychotic treatment and striatal volume increase in antipsychotic-naïve schizophrenia patients. Moreover, the treatment-induced striatal volume increase appears clinically relevant by correlating to reductions in core symptoms of schizophrenia.

## Introduction

Schizophrenia is a mental disorder affecting approximately 1% of the population worldwide (Salavati et al., 2015). The disorder typically manifests in puberty or adolescence and is characterized by so-called positive symptoms such as delusions and/or hallucinations (Howes and Kapur, 2009), but patients also exhibit negative symptoms and cognitive deficits. Studies using structural magnetic resonance imaging (sMRI) provide evidence that patients with schizophrenia display subtle volumetric brain aberrations at the time of diagnosis as compared to healthy controls (Brugger and Howes, 2017; Dietsche et al., 2017). Moreover, the brain of chronic, medicated patients appears to undergo progressive, structural changes over the course of the illness, with ventricular volume increases, cortical thinning, and basal ganglia enlargement among the most consistent findings (Brandt and Bonelli, 2008; Puri, 2010; Haijma et al., 2013; van Erp et al., 2016; Dietsche et al., 2017). Antipsychotic drugs (APD) are the gold standard for treatment of positive symptoms (Howes and Kapur, 2009), but since illness and treatment go hand in hand, separating the effects of medication and disease on brain structure is difficult (Fusar-Poli et al., 2013).

In 1976 it was discovered that antipsychotics exert their function by antagonizing the dopamine D_2_ receptors (D_2_R) in striatum, and that drug efficacy is directly proportional to the affinity for the receptor (Creese et al., 1976). This led to the dopamine hypothesis of schizophrenia, which suggests that a hyperactive striatal dopamine-system leads to ‘aberrant salience’, meaning that wrongful interpretations of harmless stimuli can eventually lead to core psychotic symptoms such as hallucinations and delusions (Kapur, 2003). Further studies of striatum have found increased presynaptic dopamine synthesis capacity and -release compared to controls, as well as higher dopamine concentrations in the synaptic cleft (Howes et al., 2009, 2012; Brunelin et al., 2013; Salavati et al., 2015). All currently marketed antipsychotics antagonize the D_2_R, thereby blocking the down-stream signaling in the post-synaptic neuron (Golan, 2012; Kusumi et al., 2015; Amato et al., 2017). However, most antipsychotics are characterized by broad receptor profiles, and bind to e.g., serotonin 2A-, histaminergic- and cholinergic receptor systems (Kusumi et al., 2015). This complex pharmacology has further limited the investigations of causal mechanisms linking antipsychotic treatment to structural brain changes. Nevertheless, longitudinal studies on antipsychotic-naïve patients as well as meta-analyses studies have reported associations between antipsychotic exposure and volumetric increase in basal ganglia (Glenthoj et al., 2007; Ebdrup et al., 2013; Jorgensen et al., 2016; Huhtaniska et al., 2017; Di Sero et al., 2019). Studies on rodents have replicated the basal ganglia volume increase in response to antipsychotic treatment (Vernon et al., 2012), and investigations in dopamine D_2_ or D_3_ receptor knock-out- and wild-type mice provide evidence that this increase is likely to be mediated through D_2_-like receptors (Guma et al., 2018, 2019).

In humans, dopamine D_2_-like receptor availability and blockade following antipsychotic treatment can be investigated with single-photon emission computed tomography (SPECT) examinations (Salavati et al., 2015). The association between antipsychotic treatment, dopamine D_2/3_ receptor occupancy, and basal ganglia enlargement has, however, yet to be established in a longitudinal study of antipsychotic-naïve patients with schizophrenia.

To address this gap in our knowledge, we completed a prospective study, wherein we examined a cohort of first-episode, antipsychotic-naïve schizophrenia patients, before and after 6 weeks of treatment with amisulpride, a relatively selective dopamine D_2/3_ receptor antagonist. Baseline- and follow-up examinations included sMRI, SPECT, and Positive and Negative Syndrome Scale (PANSS) examinations.

We hypothesized that selective blockade of dopamine D_2/3_R would lead to a dose-dependent striatal volume increase. Further, we explored correlations between symptom severity decrease, striatal volume increase, dose and receptor occupancy.

## Materials and Methods

### Participants

We included participants between the ages of 18–45 years from 2008 to 2014. Patients with schizophrenia were first-episode, antipsychotic-naïve, and were recruited from hospitals and psychiatric out-patient clinics in the capital region of Denmark, as a part of the PECANS I (Pan European Collaboration Antipsychotic-naïve Studies, PECANS) cohort. All patients met the International Classification of Diseases (ICD-10) criteria for schizophrenia (F20) verified by the structured diagnostic interview SCAN (Schedule of Clinical Assessment in Neuropsychiatry, version 2.1). Exclusion criteria included previous exposure to antipsychotic medication, methylphenidate, or use of antidepressants less than 1 month prior to baseline examinations. Healthy controls were recruited through advertisement, and matched to patients on age, gender and parental socioeconomic status. Exclusion criteria for the healthy controls were identical to the criteria for patients, but also comprised any former or current psychiatric illnesses, psychiatric diagnoses within first-degree relatives and/or any drug-abuse (classified by ICD-10). For all participants, previous or current medical history of serious head trauma, neurological diseases, developmental disorders or current drug dependency (by ICD-10 classification), and current pregnancy were exclusion criteria. All participants were screened for drug-use with urine samples (Rapid Response, Jepsen HealthCare) prior to SPECT scan. Included participants are a subsample of Wulff et al. (2015, 2019) from the PECANS I cohort. Wulff and colleagues also reported on binding potentials in their sample, although a different method of binding potential extraction was used. Subcortical volumes have not yet been investigated in this subgroup.

### Medication

The atypical APD, amisulpride, was chosen as a tool compound because of its relative selectivity toward dopamine D_2/3_ receptors (Rosenzweig et al., 2002). Amisulpride treatment was initiated after completion of baseline examinations, and dosage was slowly increased and adjusted to the individual patient, according to clinical judgment and patients’ reports of adverse effects. Pharmaceutical treatment against adverse effects was not allowed. Follow-up examinations were conducted after six weeks, and treatment dose in mg was recorded. To ensure a steady concentration at examinations, dosage was kept stable in the week prior to follow-up. Compliance was continuously ensured through dialogue with the patient, and measurement of serum-amisulpride (S-amisulpride) levels at follow-up. Benzodiazepines were allowed on an “as-needed basis” to secure sleep and reduce anxiety but were not allowed 12 h prior to SPECT examinations. Healthy control subjects were not treated.

### Symptom Severity

Symptom severity was assessed with PANSS (Kay et al., 1987) within the same week as MRI and SPECT scan examinations. PANSS total score as well as sub-scores (positive-, negative-, and general sub-scores) was assessed at baseline and at follow-up. To ensure consistency in PANSS ratings between clinicians, ratings were regularly evaluated using systematic video recordings of the interviews. Duration of untreated illness was assessed from the patient history of worsening in functions due to symptoms. Healthy controls did not undergo PANSS examinations.

### Magnetic Resonance Imaging

**FIGURE 1:**
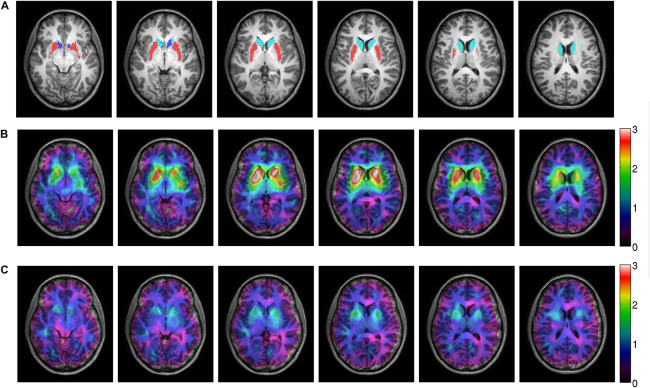
MRI and SPECT images of one patient, treated with 300 mg amisulpride displaying a mean dopamine D_2_ receptor occupancy of 56%. Panel **(A)** shows the sMRI image with the caudate nucleus (light blue), putamen (red) and accumbens (dark blue) from the subcortical Harvard-Oxford atlas depicted. Panels **(B,C)** show the co-registered SPECT image on top of the MRI image. The color scale corresponds to the specific binding potential before treatment **(B)** and after six weeks of treatment **(C)**.

T1-weighted scans of the whole head (sagittal 3D sequence, TR = 10 ms, TE = 4.6 ms, FA = 8°, voxel size = 0.79 mm × 0.79 mm × 0.80 mm) were acquired with an 8-channel SENSE head coil on a 3T Philips Achieva scanner (Philips Healthcare, Best, Netherlands) at baseline and after 6 weeks. MRI scans were acquired within the same week as SPECT and PANSS. Subcortical segmentation and volume extraction were performed with tools from the FSL, FMRIB software library v5.0.10 (Patenaude et al., 2011). In this study we focused on striatum as our region of interest, estimated as a sum of volumes from the bilateral subregions of caudate nucleus, putamen and nucleus accumbens (Figure 1). Anatomically, striatum is also referred to as a part of basal ganglia (Waschke and Paulsen, 2011).

### Single Photon Emission Computed Tomography

Single-photon emission computed tomography acquisition has previously been described (Wulff et al., 2015). In short, SPECT images were acquired using a Siemens Symbia T2 series SPECT-CT scanner, with the [^123^I]-Iodobenzamide ([^123^I]-IBZM) as the radioactive ligand, because of its dopamine D_2/3_R selectivity (Kung et al., 1990; Barnas et al., 2001). After 180 min of rest, a CT scout and 2 × 30 min tomography were performed. CT-scout and tomography were performed to optimize positioning in the scanner and for attenuation correction. Patients underwent both baseline and follow-up SPECT scans, whereas controls only underwent baseline SPECT to minimize their exposure to radiation. At follow-up, the individual dose of amisulpride was administered 3 h prior to the scan, and s-amisulpride was measured prior to and at 60, 120, 150, 180, 210, and 240 min after administration. The mean s-amisulpride during SPECT-scan was calculated.

### Image Processing

Because SPECT images contain limited anatomical information, it was not possible to automatically extract SPECT counts (counts/s) directly from our regions of interest. First, we co-registered CT- and MR anatomical images using a statistical parametric mapping method (SPM8) to calculate the transformation matrix. This step was visually inspected with an image overlay method, and manually adjusted if needed (Willendrup et al., 2004). Next, the CT-MR transformation matrix was used to co-register the SPECT images to the MRIs, and FSL subcortical region segmentations were resliced to fit the individual SPECT images. Subsequently, SPECT counts were extracted from FSL-MRI defined regions.

Lastly, extracted SPECT counts were scatter- and decay corrected. The specific binding potentials were calculated by subtracting non-specific binding from a reference region from total binding in the regions of interest divided by the metabolite corrected plasma counts. Cerebellum as defined in Svarer et al. (2005) was used as reference region for non-specific binding, as in our previous study on binding potentials (Wulff et al., 2015). Dopamine receptor occupancy was calculated using the following equation:

$$Occupancy\left(\%\right)=\left(1-\frac{S\,p\,e\,c\,i\,f\,i\,c\,b\,i\,n\,d\,i\,n\,g\,p\,o\,t\,e\,n\,t\,i\,a\,l\,\left(f\,o\,l\,l\,o\,w\,\text{-}\,u\,p\right)}{S\,p\,e\,c\,i\,f\,i\,c\,b\,i\,n\,d\,i\,n\,g\,p\,o\,t\,e\,n\,t\,i\,a\,l\,\left(b\,a\,s\,e\,l\,i\,n\,e\right)}\right)\times 100\%$$

### Statistical Analyses

Statistical analyses were conducted using IBM SPSS version 25. Normal distributions were assessed by Shapiro–Wilk. Equality of variance was assessed by Box’s- or Levene’s test. For between-groups comparisons, unpaired students t-test was used for normally distributed data and Mann–Whitney for non-normally distributed data (demographics, volumes, and binding potentials). Within-group comparisons were analyzed with paired students t-test and Wilcoxon for non-normally distributed data. Cohens *d* was used to calculate effect sizes, with effect size 0.2 considered low, 0.5 considered medium and 0.8 considered high. Pearson’s Chi^2^ was used for nominal data. When correlating data, Pearson’s correlation coefficient was used for parametric data, otherwise Spearman’s rho was used.

Our primary hypothesis was tested in two steps. First, striatal volume changes over time were tested with a repeated measure analysis. Significant group-by-time interactions were further investigated with *post-hoc* t-tests. The repeated measures analysis was initially performed for striatum, and afterward we separately analyzed the striatal subregions, i.e., caudate nucleus, putamen and nucleus accumbens. Second, we applied a multiple regression analysis to investigate the individual predictive effect of a set of variables on striatal volume increase, whilst controlling for the following included variables: amisulpride dose, striatal receptor occupancy, and baseline PANSS positive score. PANSS baseline positive scores were included in the model to control for the disease severity. Assumptions of normal distribution and no multicollinearity (Variance Inflation Factor <10) were met. If variables were initially non-normally distributed, they were transformed to normal distributions using log10- or square root functions.

Finally, we explored Spearman correlations between changes in symptom severity, striatal volumes, amisulpride dose, and D_2/3_R occupancy. Explored correlations were Bonferroni corrected for number of hypotheses tested on the same data, with a threshold of α/m, where α-level was set at 0.05, and m was number of hypotheses tested. For all other analyses, a two-sided *p*-value less than 0.05 was accepted as significant.

## Results

### Patients Compared to Healthy Controls

We included 21 patients and 23 controls with full datasets in our analyses (Supplementary Figure S1). Patients had higher use of tobacco and fewer years of education compared to controls (Table 1) but did not differ in other demographic factors. No difference in mean striatal volumes between patients and controls was found at baseline (*p* = 0.82) or at follow-up (*p* = 0.28). No difference in mean specific binding potentials to dopamine D_2/3_R was found between patients (2.49 ± 0.82) and controls (2.68 ± 0.71) (*p* = 0.25).

**TABLE 1:** Demographic and clinical data.

Between-groups	Group; mean ± SD [mean]		
	Patients (*n* = 21)	Controls (*n* = 23)	*p*-value
Demographics			
Age, years	23.5 ± 4.8	24.1 ± 5.01	0.92^b^
Sex, male:female	10:11	12:11	0.76^c^
Handedness, right:ambidextrous:left	16:3:2	20:2:0	0.31^c,f^
Handedness score,−100:100	59.2 ± 60.7	54.6 ± 68.7	0.78^b^
Parental socioeconomic status, high:moderate:low	4:11:6	5:14:4	0.68^c,f^
Educational level, higher education/self employed, medium education, uneducated, student	0:3:4:9	0:2:0:15	0.06^c,f^
Years of education	11.9 ± 2.0	14.3 ± 2.5	**0.001**^a^
Weight, kg	78.5 ± 20.6	68.5 ± 11.0	0.058^a^
Height, cm	172.8 ± 9.5	175.1 ± 10.3	0.54^a^
Substance use, alcohol, tobacco, cannabis, benzo, opioids, stimulants	16:13:4:0:1:3	20:3:1:0:0:0	**<0.001**^c,e,f^
Volumes (cm^3^)			
Baseline	18.31 ± 2.3	18.04 ± 2.5	0.82^b^
Follow-up	18.67 ± 2.3	17.92 ± 2.3	0.28^a^
Specific binding potentials (counts/s)			
Baseline	2.49 ± 0.82	2.68 ± 0.71	0.25^b^
Follow-up	1.38 ± 0.68	–	–

**Within patients**	**Baseline**	**Follow-up**	

PANSS scores^d^			
Positive	19.8 ± 4.0	13.4 ± 3.4	**<0.001**
Negative	18.7 ± 7.2	20.3 ± 5.8	0.081
General	40.1 ± 8.5	30.2 ± 7.5	**<0.001**
Total	78.5 ± 16.4	64.0 ± 13.8	**<0.001**
Medication			
Dose amisulpride (mg/day)	–	233.3 ± 109.9	
S-amisulpride (ng/ml)	–	399.7 ± 283.8	
Duration of untreated illness (weeks)	80.8 ± 96.2	−	
Receptor occupancy			
Striatum	−	44.65% ± 18.7%	

*SD, standard deviation. ^*a*^t-test, ^*b*^Mann-Whitney U, ^*c*^Pearsons Chi, ^*d*^Wilcoxon, ^*e*^*p* < 0.05 only for tobacco use, ^*f*^Groups have expected counts less than 5. Significant *p*-values are in bold.*

### Symptom Severity and Receptor Occupancy in Patients After Treatment

After six weeks of treatment, patients’ PANSS total-, positive- and general symptom scores were significantly decreased, but negative symptoms were not (Table 1). Patients were treated with a mean dose of 233.3 (SD = 109.9) mg amisulpride. Oral dose and s-amisulpride correlated positively (*r*^2^ = 0.76, *p* < 0.001). Mean receptor occupancy was 44.65% (SD = 18.7%) and correlated positively with oral dose (*r*^2^ = 0.60, *p* = 0.004) and s-amisulpride (*r*^2^ = 0.68, *p* = 0.001). Receptor occupancy is illustrated in Figure 1. Amisulpride dose did not correlate with symptom severity (PANSS total) at baseline (*r*^2^ = 0.292, *p* = 0.199).

### Striatal Volume Increase Is Predicted by Amisulpride Dose, But Not D_2/3_R Occupancy

**FIGURE 2:**
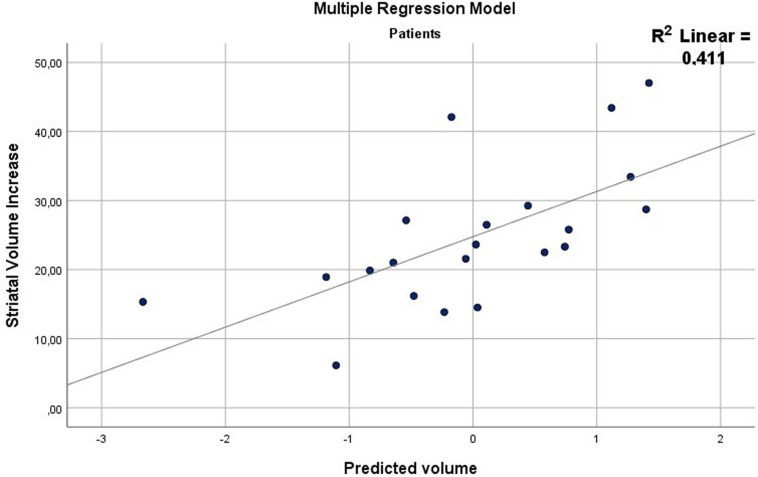
Scatter plot of the multiple regression model including dose, striatal receptor occupancy and PANSS positive score as predictive/independent variables. The dependent variable, striatal volume increase, is depicted on the y-axis, the independent variables on the x-axis. The model significantly predicted striatal volume increase (*r*^2^ = 0.411, *p* = 0.026). Only dose was a unique predictor of volume increase when controlling for the other variables (*r*^2^ = 0.553, *p* = 0.028). All model coefficients can be seen in Supplementary Table S1.

The repeated measures analysis revealed no volume difference between groups at either time-point, but instead a significant group-by-time interaction was observed (*p* = 0.01). The *post hoc* analysis revealed that the interaction was driven by a significant volume increase in striatum of 2.1% (95% CI = 0.52–3.68%, *p* = 0.01, Cohens *d* = 0.45) in patients. Sub-regional increases were observed in left and right caudate nucleus (2.6%) and right putamen (2.4%) (Table 2). The multiple regression model significantly predicted striatal volume increase (*r*^2^ = 0.411, *p* = 0.026) (Figure 2), with amisulpride oral dose as the only unique, predictive factor (beta = 0.553, *p* = 0.028) (Supplementary Table S1).

**TABLE 2:** Volumes of regions of interest.

Volume (cm^3^)	Patients			Controls		
	Baseline mean ± SD [mean]	Follow-up mean ± SD [mean]	*p*-value	Baseline mean ± SD [mean]	Follow-up mean ± SD [mean]	*p*-value
Striatum	18.31 ± 2.3	18.67 ± 2.3	**0.01**	18.04 ± 2.5	17.92 ± 2.3	0.121^a^
Caudate	7.68 ± 1.1	7.88 ± 0.1	**<0.001**	7.45 ± 0.9	7.37 ± 0.8	0.187
Left	3.74 ± 0.5	3.88 ± 0.5	**0.003**	3.68 ± 0.4	3.61 ± 0.4	0.067
Right	3.94 ± 0.6	4.00 ± 0.6	**0.004**	3.77 ± 0.5	3.76 ± 0.4	0.770
Putamen	9.66 ± 1.3	9.82 ± 1.3	**<0.001**	9.63 ± 1.6	9.66 ± 1.5	0.224^a^
Left	4.87 ± 0.7	4.91 ± 0.6	0.347	4.81 ± 0.9	4.88 ± 0.9	0.670^a^
Right	4.79 ± 0.6	4.91 ± 0.7	**0.007**	4.82 ± 0.8	4.80 ± 0.7	0.212^a^
Accumbens	0.97 ± 0.2	0.98 ± 0.2	0.732	0.96 ± 0.2	0.97 ± 0.2	0.484^a^
Left	0.53 ± 0.1	0.55 ± 0.1	0.627	0.55 ± 0.1	0.55 ± 0.1	0.879^a^
Right	0.43 ± 0.09	0.43 ± 0.08	0.614^a^	0.41 ± 0.1	0.42 ± 0.1	0.346^a^

*Equal variances were tested. *p*-values marked with ^*a*^ was tested with Wilcoxon. All other volumes were tested with paired students *t*-test. Significant *p*-values are in bold.*

### Symptom Severity Exploratory Correlations

Reduction in positive symptoms correlated significantly with striatal volume increase (*r*^2^ = −0.472, *p* = 0.031) and this correlation was driven by a reduction in hallucinations (*r*^2^ = −0.515, *p* = 0.017). The correlations did not survive Bonferroni correction. Changes in PANSS total- or subscores did not correlate to either amisulpride dose, s-amisulpride or receptor occupancy.

## Discussion

### Primary Findings

In line with our hypothesis, we found a significant volume increase in striatum in patients (2.1%) with a medium effect size (Cohens *d* = 0.45) after six weeks of amisulpride treatment. Our predictive model showed that dose was a predictor of volume increase, but positive symptom severity at baseline and D_2/3_R occupancy were not. Our exploratory correlation analyses indicated that striatal volume increase was associated with an improvement in positive symptoms, particularly hallucinations.

### Results Compared to Previous Findings

Structural brain differences between patients and healthy controls at the time of diagnosis have previously been reported, but are not consistently replicated, and often no differences are found, indicating that changes are subtle (Glenthoj et al., 2007; Puri, 2010; Dietsche et al., 2017). Consistent with this, we did not find any significant differences in striatal volumes between patients and healthy controls. Specific D_2/3_R binding potentials did not differ between patients and controls prior to treatment, which is a replication of previous findings (Howes et al., 2009; Salavati et al., 2015). In this study, mean amisulpride dose was relatively low (233.3 mg), and approximately half of the dose used in phase one of the OPTiMiSE study (488.0 mg for completers) (Kahn et al., 2018). However, included patients in the OPTiMiSE study were not all antipsychotic-naïve, and due to a potential compensatory upregulation of D_2_R in response to antipsychotic treatment (Oda et al., 2015; Yin et al., 2017), this could explain the need for higher treatment doses compared to our antipsychotic-naïve patients. Furthermore, medication against adverse symptoms was not allowed in this study, which made clinicians upregulate dose slowly.

Treatment dose and blood levels are inherently linked to occupancy, and it is generally accepted that a striatal dopamine receptor occupancy of 65–80% is necessary for clinical response (Uchida et al., 2011; Yilmaz et al., 2012). However, we found a significant decrease in symptom severity at mean receptor occupancy-levels of 44.65%. Most recent studies investigating occupancy levels in patients use estimations, but a cautious comparison can be made to the CATIE data (Moriguchi et al., 2013). Authors found that for patients in stable remission, approximately half did not have continuous dopamine D_2_ blockade of ≥65%. A prospective PET study further found an optimal therapeutic window between 50 and 60% receptor occupancy on clinically stable patients with late-life schizophrenia (Graff-Guerrero et al., 2015). Altogether, this indicates that lower doses/D_2_ receptor occupancies in selected patient populations are sufficient, and also reduce risk of adverse effects such as extrapyramidal symptoms.

We found no correlation between reduction in positive symptoms and dose or occupancy as previously found for amisulpride (Sparshatt et al., 2009) and other antipsychotics (Yilmaz et al., 2012), but a negative finding has also been reported (Batail et al., 2014). Different patient groups, different antipsychotics and different methodology makes results difficult to compare. Considering that amisulpride in low doses has a higher affinity toward the presynaptic dopamine D_2_ auto receptors rather than post synaptic receptors (Rosenzweig et al., 2002), the discrepancy may be explained by the relatively low dose used in our study. We did, however, find a correlation between symptom reduction and volume increase. When specific positive symptoms were examined, this correlation was linked to a decrease in hallucinations. Similar results were found in a study by Li et al. (2012), in which PANSS decrease correlated to volume increase in putamen. Our results did not survive a Bonferroni correction and should be interpreted with caution. However, this plays well into hypotheses related to altered striatal structure and connectivity linked to symptom severity (Sarpal et al., 2015).

### Basal Ganglia Volume Increases

Correlation between antipsychotic dose and volume changes in striatum is a subject of much debate (Roiz-Santianez et al., 2015; Huhtaniska et al., 2017) in part because separating the effect of disease and medication is inherently difficult. Vernon and colleagues found proof of concept in healthy rodent models, in which chronic (8 weeks) exposure to antipsychotics, but not other psychotropics (e.g., lithium) using clinically comparable dosing, leads to structural brain changes in naïve rats, including striatal enlargement (Vernon et al., 2012). In line with these data, we found a dose-dependent volume increase in the striatum after six weeks of treatment with an atypical APD with predominant D_2/3_R blockade. It is still, however, unknown what causes this volume increase. Investigation has been made into the cellular components of the volume increase, but linking structural MR changes to their cellular correlates is challenging, and although antipsychotic exposure has been found to moderate microglial activation, neuronal dendritic spine density and astrocytes (Vernon et al., 2014; Cotel et al., 2015; Amato et al., 2017), no studies to date have linked any of these changes to striatal volume increase.

Another explanation for the volume increase could be augmented blood flow to striatum. This was found in a functional MRI study in healthy males after one dose of APD (Hawkins et al., 2018), as well as in patients treated with a mean of 27 days (Corson et al., 2002). Increased blood flow could possibly lead to an “apparent” volume change, but the difference in flow did not seem to have an impact on volume changes or brain structure investigated by Hawkins et al. (2018). Notably, Vernon et al. (2012) reported that striatal volume increases in rodents chronically exposed to haloperidol (8 weeks) was normalized after an equivalent period of drug washout (Vernon et al., 2012). The same tendencies of volume decrease in putamen after withdrawal of antipsychotics was reported from a small schizophrenia patient cohort (Boonstra et al., 2011). Finally, our previous functional MRI study on a subset of the current cohort showed changes in the task-related blood oxygen level-dependent activation in striatal regions after amisulpride treatment (Nielsen et al., 2012). Collectively, these data suggest that the effects of antipsychotics on brain structure, including the basal ganglia, are dynamic and potentially reversible.

It has long been discussed whether striatal volume changes are specific to so-called typical antipsychotics (Ebdrup et al., 2013), but our findings together with (Jorgensen et al., 2016) and (Glenthoj et al., 2007) show that this is not the case. The assumption may have been due to striatal volume decreases seen in patients treated with atypical clozapine (Garcia et al., 2015; Jorgensen et al., 2016) or quetiapine (Ebdrup et al., 2011). Both drugs, however, have low affinity toward dopamine D_2_-like receptors, whereas typical antipsychotics have high affinities (Creese et al., 1976; Kusumi et al., 2015; Jorgensen et al., 2016). Dopamine D_2_-like receptor knock-out mice also show striatal volume increases, mirroring the effects of chronic exposure (9 weeks) to different APDs (Guma et al., 2018, 2019). Notably, when chronically exposed to the same antipsychotics as wild-type mice, no additional basal ganglia volume increases were found. Taken together, these data strongly support that volume increases following antipsychotic exposure are mediated via the dopamine D_2_-like receptor (Guma et al., 2018, 2019), rather than depending on the drug-class (‘typical’ vs. ‘atypical’ antipsychotic).

### Dopamine Receptor Occupancy

We expected the volume increase to be predicted by striatal dopamine D_2/3_R occupancy, because occupancy may be considered a more direct measure of effect than oral dose. This was not the case. We speculate that it may be due to several issues regarding SPECT imaging. First, receptor occupancy is calculated as the difference in available dopamine receptors between baseline and follow-up, and is therefore subjective to interfering factors such as changes in endogenous dopamine levels, which in turn affect dopamine receptor availability and occupancy. Second, as previously mentioned, studies also suggest a possible compensatory upregulation of D_2_R in response to treatment (Oda et al., 2015; Yin et al., 2017), which again may affect occupancy, and a potentially decreased effect of drug dose in the long-term. Third, SPECT measurements are subjective to noise, which may have obscured a potential true correlation. Another issue to consider is that the multiple regression analysis assumes linearity, which might not be the case between volume increase and receptor occupancy. Lastly, a measure of cumulative dose (although likely correlated to the mean dose) might have been a more accurate measure, but unfortunately not possible within our study. To our knowledge, only one study has done a similar investigation, also reporting no association between occupancy and volume increase (Di Sero et al., 2019). However, since amisulpride primarily acts by blocking D_2/3_R, we argue that the observed striatal volumetric increases still can be mediated through occupancy, and we assign the negative association with occupancy to the aforementioned issues.

### Strengths and Limitations

We conducted a clinically challenging prospective study on a cohort of antipsychotic-naïve first-episode schizophrenia patients and matched, healthy controls. Men and women were equally represented and confounding effects of previous exposure to antipsychotics could be ruled out. Amisulpride was chosen for treatment because of its selectivity toward dopamine D_2/3_R, thereby excluding potential involvement of other neuroreceptors.

Because of the extensive examination program, the study included a limited sample of patients, and therefore selection bias cannot be ruled out. On the other hand, with a mean baseline PANSS total score of 78.5, the patients in our study may be considered moderately ill (Leucht et al., 2005). The limited number of patients restricted the degrees of freedom in the multivariate linear regression model, and therefore it was not possible to include- and control for further variables in our analyses. The effect of nicotine on basal ganglia volumes is unresolved (Van Haren et al., 2010; Das et al., 2012).

## Conclusion

We found a dose-dependent striatal volume increase in antipsychotic-naïve schizophrenia patients in response to six weeks dopamine D_2/3_ receptor blockade with an atypical antipsychotic compound. Thus, our findings contrast the notion that striatal volume increase is restricted to “typical” antipsychotics. However, the underlying mechanisms warrant further investigation. We found the striatal volume increase to be clinically relevant, since it appears correlated to a reduction in positive symptoms.

## Data Availability Statement

The datasets generated for this study are available on request to the corresponding author.

## Ethics Statement

The study was conducted in accordance with the Helsinki declaration II, and approved by the Danish research ethics committee (H-D-2008-088), as well as the Danish Data Committee (RHP-2016-025, I-suite no. 05181). Clinical Trials.gov Identifier: NCT01154829. The patients/participants provided their written informed consent to participate in this study.

## Author Contributions

BG and BE conceived and designed the study. SW and MN collected the data. JR, CS, SW, ER, LJ, PA, and LP contributed with data processing and analysis tools. HA, JR, LBJ, ER and BE conducted the statistical analyses. HA and BE drafted the manuscript. HA, JR, CS, and SW particularly contributed with method section. AV contributed to the interpretation and discussion. All authors fulfill authorship criteria of the ICMJE by substantial contribution to the conception and design, to acquisition of data, or to the analysis and interpretation of the data contributed to manuscript revision, read and approved the submitted version.

## Conflict of Interest

BE has received lecture fees and/or is part of Advisory Boards of Bristol-Myers Squibb, Eli Lilly and Company, Janssen-Cilag, Otsuka Pharma Scandinavia AB, Takeda Pharmaceutical Company and Lundbeck Pharma A/S. BG is the leader of a Lundbeck Foundation Centre of Excellence for Clinical Intervention and Neuropsychiatric Schizophrenia Research (CINS), which is partially financed by an independent grant from the Lundbeck Foundation based on international review and partially financed by the Mental Health Services in the Capital Region of Denmark, the University of Copenhagen, and other foundations. Her group has also received a research grant from Lundbeck A/S for another independent investigator-initiated study. All grants are the property of the Mental Health Services in the Capital Region of Denmark and administrated by them. She has no other conflicts to disclose. The remaining authors declare that the research was conducted in the absence of any commercial or financial relationships that could be construed as a potential conflict of interest.
