# Supplementary material for: Striatal Volume Increase After Six Weeks of Selective Dopamine D2/3 Receptor Blockade in First-Episode, Antipsychotic-Naïve Schizophrenia Patients
Source: Front Neurosci. 2020 May 20;14:484. doi: 10.3389/fnins.2020.00484 (PMC7251943; doi:10.3389/fnins.2020.00484)
Supplement: Supplementary file 1 [file Data_Sheet_1.docx]

Supplementary Material

## Figure S1


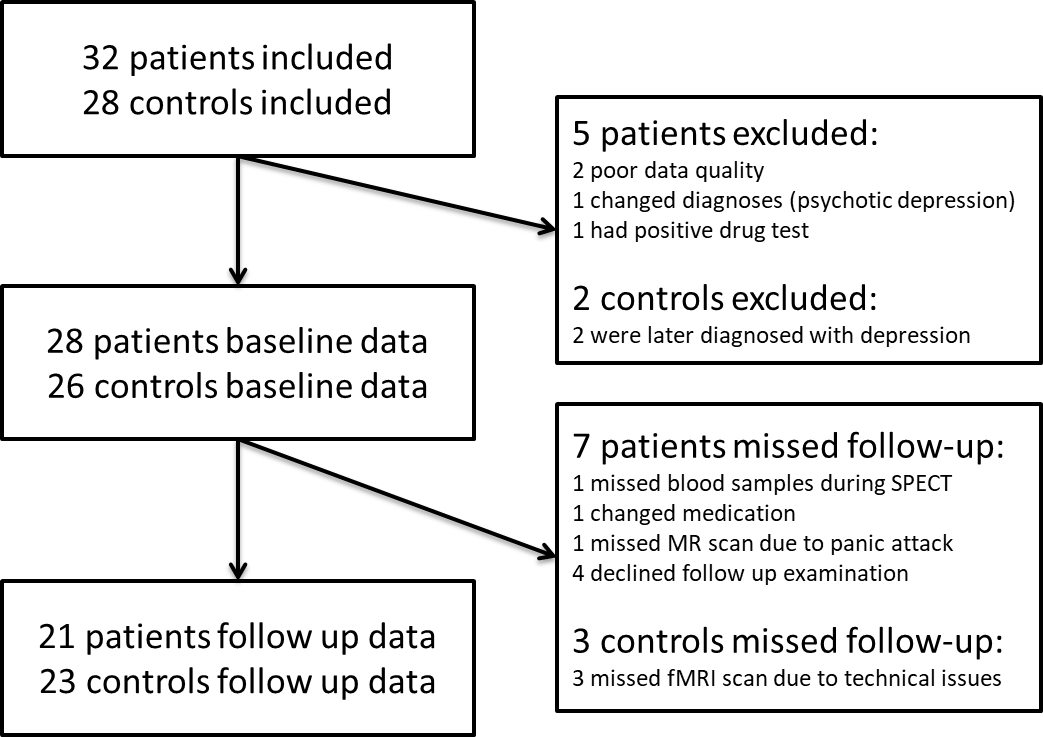


Figure S1: Consort Flow Diagram. Adapted from *(Wulff* et al.*, 2015)*.

## Table S1

| **Multiple Regression Model** | | **Standardized Coefficients** | **t-value** | **p-value** |
| --- | --- | --- | --- | --- |
|  |  | **Beta** |  |  |
|  | (Constant) |  | -.855 | .404 |
|  | Dose | .553 | 2.394 | .028 |
|  | Receptor occupancy | -.017 | -.070 | .945 |
|  | PANSS positive | .244 | 1.251 | .228 |
|  | Model regression |  |  | 0.026 |

Table S1: Multiple regression model coefficients. The model significantly predicted volume increase (p=0.026) with dose as the only unique, predictive variable (p=0.028).
